# Cannabis Use Among Mental Health Professionals: A Qualitative Study of Cannabis-Related Risk Perceptions

**DOI:** 10.1177/00220426211032558

**Published:** 2021-07-20

**Authors:** Amar Ghelani

**Affiliations:** 1 Faculty of Social Work, 8431 Wilfrid Laurier University, Toronto, ON, Canada

**Keywords:** cannabis, marijuana, risk, mental health, professionals, risk perceptions

## Abstract

Background: Perceptions of cannabis-related risk are changing, and many are viewing cannabis as harmless despite the biopsychosocial risks. Perceptions of risk have an impact on behavior as individuals who are less likely to view cannabis as risky are more likely to use it problematically. Purpose: This study examined how mental health professionals who use cannabis perceive the risks related to use. Methods: Interpretative phenomenological analysis was utilized to understand how participants made sense of the harm related to personal and client use. Interviews were conducted with a sample of social workers, nurses, and psychotherapists who work with cannabis-consuming clients. Results: Participants reported cannabis use is related to anxiety, relational challenges, impaired driving, psychosis, cognitive impairment, educational/employment dysfunction, and addiction in some users. Conclusion: Assessing risk perceptions among cannabis users can reveal subtle psychosocial problems the user may be experiencing. Mental health workers may benefit from further education regarding cannabis-related physical health harm.

## Background and Purpose

Perceptions of cannabis-related risk have been changing across Canada since cannabis legalization in 2018. Many view this drug to be relatively harmless despite the known psychological, physical, and social risks (Government of Canada, 2018; WHO, 2016). Perceptions of risk have a significant impact on drug-taking behavior as individuals who are less likely to view cannabis as risky are more likely to use it problematically (Hellemans, Wilcox, Nino, Young, & McQuaid, 2019). Furthermore, public understanding of the harm associated with cannabis can impact social stigma and access to care for cannabis users (Kerridge et al., 2017). Mental health professionals offer therapeutic services for cannabis users with biopsychosocial difficulties, although cannabis use and perceptions of risk among the professionals are under-researched areas. The purpose of this study is to examine how Canadian mental health professionals who consume cannabis perceive the risks and harms associated with this drug. Canadian professionals were targeted to better understand how they rationalize their use in relation to the perceived risks and their clients use in an environment where cannabis is legal. This group possesses insider knowledge and views that are important to educating the public and reducing stigma and barriers to care for cannabis users seeking mental health support. The findings from this study can be used to support people (including mental health professionals) seeking therapeutic support for problematic cannabis use.

Acute cannabis consumption can cause anxiety, panic, cognitive impairment, and psychotic symptoms in some users, as well as motor vehicle accidents among impaired drivers (WHO, 2016). Frequent, early, and long-term use is associated with dependence, educational challenges, persistent cognitive problems, respiratory issues, cardiovascular disease, and mental illnesses such as psychotic and anxiety disorders (WHO, 2016). Long-term use has also been associated with suicidal ideation and behavior (Gobbi et al., 2019). Canadians have indicated the major harms they associate with cannabis use are impaired driving, pulmonary damage, throat problems, and potential for dependence (Government of Canada, 2018; Hathaway, 2003). This drug is considered by many to be a less serious social problem than use of tobacco or alcohol (Cunningham & Koski-Jännes, 2019), although cannabis-related hospitalizations are now common in youth under twenty-four years (CIHI, 2019). Evidence suggests that youth aged 15–24 years are less aware of the risks related to cannabis use, particularly in relation to driving, as one in five young people who have used cannabis report driving after consumption (Wallingford, Konefal, & Young, 2019). Despite the potential harms, almost 60% of Canadian cannabis users describe it to be more helpful than harmful to their mental and physical health (Spackman et al., 2017).

Previous studies have applied rational choice theory to understand cannabis use among adult Canadians and related risk perceptions (Hathaway, 2003; Osborne & Fogel, 2008). This lens views individuals as logical agents, able to consider available information, probabilities of outcomes, and potential costs/benefits in determining their preferences and courses of action (Scott, 2000). Cannabis use can be conceptualized from this perspective as a product of an individual’s determination that the psychoactive effects of the drug are beneficial enough to justify continued consumption despite detrimental outcomes. The mental health professionals in this study have reported using cannabis to enhance relaxation, sleep, enjoyment, and social experiences while slowing thought processes and alleviating health concerns such as chronic pain (Ghelani, 2020). Reported reasons for cannabis use were comparable to surveys of general samples of adult Canadians that also identified relaxation and stress reduction as strong motivators for consumption (Deloitte, 2018; Osborne & Fogel, 2008).

Cannabis use has long been considered a means to relieve stress (Hyman & Sinha, 2009), and some researchers have conceptualized substance use among health professionals as an attempt to cope with stressful career demands (Coombs, 1996; Kenna, Baldwin, Trinkoff, & Lewis, 2011). In the context of excessive cuts to health services and exceedingly heavy workloads, these professionals may view the benefits of stress relief as more important than any risks which may be incurred (Bradley, 2018). Mental health workers are currently under compounded pressure due to the crisis triggered by the novel coronavirus pandemic (World Economic Forum, 2020). As more people seek care for problematic substance use and psychological challenges due to the pandemic, these professionals are also at elevated risk for anxiety, distress, and substance use issues (Tasker, 2020). Legal cannabis sales have spiked since governments shut down parts of the economy in March 2020 (George-Kosh, 2020), and it is possible that increasing numbers of Canadians, including some mental health workers, will find themselves coping with this unprecedented situation through problematic cannabis use.

Only 2% of lifetime cannabis consumers in Canada have sought professional support for related problems (Government of Canada, 2018). While many consumers may not require mental health support, fear of judgment and stigma are major barriers to care for those who are considering seeking help (Kerridge et al., 2017). A large-scale survey of non-users reported 54% believe there is still negative stigma associated with this drug (Caddle, 2018). Health professionals may experience additional stigma as they are typically known for offering help rather than receiving it (Bradley, 2018). According to the Centre for Addiction and Mental Health, stigma can be challenged through knowing the facts, dispelling myths, educating others, being inclusive, and talking carefully about mental health and substance use (CAMH, 2020). The findings from this study will reduce stigma and barriers for cannabis users (including health professionals) who may be considering accessing mental health support through illuminating the knowledge of professionals with insider perspectives.

## Methods and Procedures

This study followed a qualitative design informed by interpretative phenomenological analysis (IPA). This approach involves exploring perspectives, meanings, and lived experiences of a specific phenomenon, with emphasis on understanding how participants make sense of their personal and social worlds (Smith, Jarman, & Osborn 1999; Smith & Osborn, 2008). Interpretative phenomenological analysis can illuminate the complexity of psychosocial phenomena while offering transformative possibilities for informing clinical practice. In the IPA methodology, awareness of researcher preconceptions and assumptions are fundamental to interpreting participant responses (Moustakas, 1994; Smith et al., 1999).

Semi-structured interviews were conducted with a sample (*n* = 7) of Canadian mental health professionals. Convenience and purposive sampling were utilized. Eligible participants (a) were regulated mental health professionals, (b) had direct service experience with cannabis-using clients, and (c) had recreationally consumed cannabis at least once in the past month. The sample consisted of three registered social workers, two nurses (one nurse practitioner), and two registered psychotherapists. The four female and three male professionals reported employment in community mental health, hospital, and private practice settings. They described serving clients including children, youth, families, and adults with a broad range of psychosocial challenges. The study’s methods were approved by a Canadian University Research Ethics Board (REB), and participants provided written informed consent to participate in the study. Guidelines proposed by the REB limited questioning participants around negative personal experiences with cannabis to prevent undue distress. Additional consideration was given to privacy and confidentiality due to the sensitive nature of the subject matter. Participants were de-identified in all possible documentation and given an opportunity to review the findings to ensure quotes could not reveal their identities (Sinding, Gray, & Nisker, 2008). Interview questions were open-ended and focused on motivations for cannabis use, perceptions of risk, and views on client use.

Coding involved reading across interviews using NVIVO software and identifying salient statements that reflected shared perceptions of risk, harm, or unwanted cannabis effects (Padgett, 2017; Smith et al., 1999). Once *risk codes* were identified for each interview, interrelated codes were separated and categorized into distinguished clusters based on similarities in language and meaning. Clusters with overlapping content and meanings were then grouped into new nodes to form encompassing themes. The themes were prioritized according to the quantity of shared experiences and codes, with themes noted by less than four participants discarded. Participants were offered an opportunity to review the findings and provide feedback to “respect the sensibilities of the people being represented” and reduce the risk of misrepresentation (Sinding et al., 2008, p. 465).

Analysis focused on connecting, contrasting, and contextualizing the findings (Li & Seale, 2007; Smith & Osborn, 2008). Connections between the themes were explored to understand how the various risks and harms interact to affect the mental health, relationships, and functioning of cannabis users. Themes were also contrasted with findings from related research on motivations for cannabis use, with rational choice theory applied to interpret how participants explained their choice to use cannabis despite awareness of potential harm. Findings were situated in the broader social context through linking themes with relevant research noted in the literature review.

## Results

“[Cannabis] should be treated with caution and care … I think that we have a long way to go before it finds its proper place in our culture” – Participant 7

Psychosocial risks related to cannabis use were described by all seven participants. Six stated they had encouraged clients to reduce or stop using at some point. Findings will note where participants felt particular risks were relevant to their personal use rather than use by their clients.

### Anxiety and Avoidance

The potential for cannabis to cause or exacerbate anxiety, panic, social anxiety, and/or avoidance was the most prevalent risk identified by six participants. Participant 5 stated: “Anxiety is probably experienced a little heavier” while under the influence of cannabis. This was especially concerning for youth and novice users because, according to Participant 5, “they’re not used to it” and they may not be expecting the drug to trigger anxiety-related sensations. According to Participant 5: “When I used to see people come into ER [after consuming cannabis], a lot of them came in with, like, a ‘bad trip,’ but they were just experiencing a lot of anxiety.” Cannabis was also reported by Participant 2 to make it “hard to cope” with feelings of anxiety and depression. Two participants (2 and 6) disclosed cannabis personally increased their anxiety at times, with Participant 6 stating: “I don't really smoke in public nowadays ‘cause it kind of gives me social anxiety.” Anxiety is clinically related to avoidance, and the potential for cannabis to be used to “avoid painful emotions” and “not allow [clients] to really examine what they’re feeling” was also discussed by Participants 1 and 7.

### Relational Challenges

Six participants described how cannabis could negatively affect interpersonal relationships. Four reported it could impair one’s “ability to function [and] socially cope” by “limit[ing] your ability to interact with others, ‘cause you can get panicky” [Participant 2]. Participant 7 explained: “I had young clients… getting high with their friends because that’s what everyone’s doing. But they’re having a horrendous, horrific experience while everyone else is enjoying it. But they don’t want to say ‘no’ because of peer pressure.” It was noted by Participant 2 that some clients are reluctant to accept recommendations to reduce or discontinue use because they fear “they’re going to lose connection[s]” with peers. Three informants expressed concerns about the potential for cannabis to “cause some tension in a relationship… if a parent or a spouse really feels strongly that it is not a good thing to be using” [Participant 2]. According to one professional who works with youth:

A lot of parents have rules in their home of not using, or kids aren’t allowed in if they’re clearly high … it makes the other parts of just living more challenging. So if they’re not allowed home on Sunday night because they’re clearly high, then it’s harder to have somewhere to sleep at night, and a meal that evening, and get … to school the next day. [Participant 3]

A component of relational challenges identified by participants 4 and 5 was “social stigmatization.” Negative assumptions about the effects of cannabis can result in judgments toward users being “criminal[s]” or “lazy” [Participant 4]. These social perceptions were reported to contribute to self-stigma: “Some people say, like, they’re just getting known as ‘the stoner.’ So, they do have a label of ‘this is who I am.’ … I do wonder [how] that impacts them and how they view themselves” [Participant 2].

### Cannabis-Impaired Driving

Another common risk identified by the professionals was the potential for cannabis-impaired driving.^1^ Six participants described “driving while high” as risky, with Participant 6 stating: “Being stoned and driving is obviously a huge no-no.” This behavior was also described in relation to “chronic” use and employment problems as some workplaces require operation of a vehicle and the harm associated with impaired driving can increase when it occurs while at work.

### Psychosis and Schizophrenia

Five participants reported cannabis use “could lead to psychosis” or exacerbate symptoms of schizophrenia. According to Participant 5, “There are certain people that … I’ve recommended probably should stop [using cannabis] because … they were experiencing symptoms that …fell along the schizophrenia [spectrum].” Participant 4 stated: “I’ve had clients where doctors identified marijuana as being the reason for [developing] schizophrenia.” The potential for cannabis to make young people more vulnerable to psychosis or long-term users more resistant to antipsychotic treatment was also discussed.

### Cognitive Impairment

Five participants described how cannabis could affect various dimensions of cognitive functioning. Four expressed concerns about the potential for disrupted adolescent brain development: According to Participant 2: “If they’re under the age of 25, there’s some pretty good literature on brain development in teenagers. So right away, if you’re under 25, I’m going to be giving some education on what happens [to] the brain [while using cannabis].” Three informants described how it could negatively affect attention and concentration, with Participants 5 and 6 personally disclosing: “it decreases my concentration” and “if I smoke too much, my mind starts to wander.” Participants 4 and 7 discussed how cannabis could contribute to “decreased motivation” and reduced “ability to function on a day-to-day basis.” Concerns about impaired memory were noted by Participants 2 and 1, particularly in relation to “retaining information” and “remember[ing] key facts when they’re starting a new role” at work or school. One professional [Participant 6] shared the story of a client with an acquired brain injury who struggled with “managing his confusion … just remembering things was an issue. But when he stopped smoking [cannabis], you know, those issues kind of, like, went away.”

### Education and Employment Dysfunction

The potentially deleterious effects on academic and employment functioning were noted by five participants. Concerns were expressed regarding cannabis impacting clients’ ability to secure, prepare for, and maintain employment. According to Participant 1: “I have had clients that are using daily that I’ve encouraged to reduce their use, especially surrounding when they’re going to be starting a new job.” Others also shared concerns about clients and members of the public “smoking at work,” with Participant 6 explaining, “if they’re chronic [users] then it can affect their ability to work … operate a vehicle [and] interact with others… you forget things, obviously, when you’re at work and stoned.” Several professionals described how cannabis could affect academic functioning, with Participant 3 stating: “Some [students] are skipping school to go and smoke… [then] they have a harder time focusing or, like, being an active participant in school.”

### Habit, Dependence, and Addiction

The potential for cannabis consumption to become a “chronic” habit or addiction was noted by four participants. According to Participant 6: “the risk for addiction is very real… it can be really, really hard to stop.” Participant 7 expressed concerns about clients developing “negative, unhealthy pattern[s]” of consumption or needing to “wake and bake” (smoke after waking in the morning) as signs of dependence. Two participants were personally concerned about developing a “psychological addiction.” Participant 6 shared: “There are times when I tell myself, oh, I shouldn’t do it, but I do it anyway, you know? Um, so it was prominently habitual… sometimes I regret smoking or smoking so much.” Participant 2 shared that sometimes after using it to manage physical or psychological discomfort:

I’m really pissed off at myself that I couldn’t [cope] myself and make myself feel better… I’m not using cannabis to get blasted and completely detach. I’m using it … to calm [myself] when I can’t use the available skills in that moment.

## Discussion

The risks identified by the participants in this study are interconnected and reflect how the harms associated with cannabis use impact one another. The potential for this drug to cause or worsen anxiety and psychotic symptoms in some users was noted by most participants, with these effects reportedly contributing to relational difficulties. On occasions when social anxiety or panic is felt after taking cannabis, the consumer may find it difficult to communicate effectively or navigate complex social situations. Difficulties managing stressful interactions can intensify interpersonal anxiety, at times prompting increased cannabis use in an attempt to reduce or avoid that feeling. A cycle of cannabis causing anxiety and anxiety leading to more cannabis use may ensue, with the drug being consumed to manage a condition it may be aggravating. As noted by one participant, the experience of cannabis-induced panic in a social situation where others appear to be enjoying themselves can be isolating, although peer pressure creates barriers to limiting consumption. For those who belong to social circles where cannabis is common, recommendations from professionals to reduce or stop may be met with ambivalence, reluctance, or resistance. This reality produces barriers to treatment for individuals whose anxiety escalates to psychotic symptoms such as paranoia or perceptual disturbances. Cannabis-induced psychosis, panic, and/or “bad trips” can result in hospitalization while wreaking havoc on one’s social and family life.

Several participants linked the themes of cognitive impairment and addiction to problems with school, employment, and driving. When cannabis intake becomes a habit or dependence, changes to memory, motivation, attention, and concentration can become entrenched (WHO, 2016). These diminished capacities affect a student’s ability to focus in class, retain information from lessons or readings, recall facts for tests, set goals for the future, and take steps toward those goals. Mechanisms of anxiety and avoidance may also explain why some students who feel uncomfortable at school miss class to consume cannabis. Longitudinal studies have confirmed participant concerns that cannabis use is associated with school absenteeism, lower educational attainment, and early school dropout (Lynskey & Hall, 2000; Melchior et al., 2017). Participants also noted that the risks of operating a vehicle or attending work while impaired may be downplayed by those who use frequently. When repetitive cannabis use becomes normalized, awareness of its effects on memory, motivation, and concentration can be forgotten or ignored, leaving its potentially severe consequences internally unprocessed. Similar to education, studies have established links between cannabis and lower work productivity, job loss, and persistent unemployment (Airagnes et al., 2019; Zhang, Brook, Leukefeld, & Brook, 2016). However, many cannabis users do not experience academic or vocational difficulties and factors such as genetics, trauma, and social disadvantage can also contribute to negative educational and employment outcomes (Lynskey & Hall, 2000; WHO, 2016). Nevertheless, there is conclusive evidence that cannabis impairs driving and increases risk for traffic injuries, with dependence and cognitive disruption likely facilitating this dangerous behavior (WHO, 2016).

A personal risk/reward analysis was described by almost all the professionals in this study. While cannabis was reported to increase anxiety and hinder cognition in some users and two participants noted concerns about dependence, the majority indicated that the benefits of consumption eclipsed the costs. This group weighed the harms they knew could be incurred against the benefits they perceived and determined that consumption was a reasonable personal choice. Particularly for those managing pain or insomnia, cannabis was reported to be the best option for relief. In studies by Hathaway (2003) and Osborne and Fogel (2008), larger samples of adult Canadians similarly acknowledged the benefits of enhanced relaxation, sleep, socialization, and leisure activities as meaningful enough to use this drug despite its risks or harms. It is noteworthy that these studies were primarily conducted with moderate users who may not be experiencing severe psychosocial challenges. Adult Canadians and professionals who encounter significant negative outcomes due to cannabis use are presumably less likely to view their consumption as rational.

Two participants in this study indicated that they sometimes regretted using this drug. One described ambivalence, at times espousing the benefits and at other times wishing they had alternative skills to cope. Another alluded that the negative effects of social anxiety, sleep disturbance, and wandering thoughts overshadowed the benefits despite ongoing use. While Coombs (1996) and Kenna et al. (2011) conceptualized problematic substance use among health workers as an attempt to cope with work stressors, cannabis was only briefly linked to employment pressure by one participant. What appeared to be more consistent was the subjective and variable effects of this drug. For example, cannabis was paradoxically reported to decrease anxiety and enhance thought patterns for some while increasing anxiety and interfering with cognition in others. This suggests that the determination of cannabis use as a rational choice is individualized. Rational choice theory may be practical for understanding cannabis consumption among some adult Canadians, mental health professionals, and moderate users, although it does not adequately explain this phenomenon for those who consume despite harms outweighing benefits.

The professionals in this study demonstrated an intricate understanding of the psychological, social, familial, and functional risks associated with cannabis use. Their cumulative, off-hand knowledge reflected many of the concerns outlined in a World Health Organization report on nonmedical cannabis use (WHO, 2016). Their perspectives on risk also appear to be more expansive and nuanced than the broader Canadian public (Government of Canada, 2018; Hathaway, 2003). In line with other psychiatric specialists, the participants were careful in describing the effects of the drug and emphasized the psychosocial risks for vulnerable groups such as youth and people with mental illness (George, Hill, & Vaccarino, 2018). Although most determined consumption to be more beneficial than harmful for themselves, there was a common awareness that this drug affects people differently and certain populations are at increased risk for harm.

Interestingly, only one participant mentioned the potential for smoking cannabis to cause physiological damage. While many Canadians acknowledge the risk for pulmonary injury and medical professionals link long-term use to cardiovascular disease, none of the informants noted these harms (Government of Canada, 2018; Hathaway, 2003). This absence reflects a narrow focus among some mental health workers on psychosocial phenomena at the expense of recognizing physical harms. It is possible that the participants’ personal and clinical experiences had not sensitized them to these particular concerns, as individuals struggling with physical health problems such as lung or heart problems are more likely to consult a general practitioner or other medical specialist.

This study shed light on how Canadian mental health professionals who use cannabis can reduce stigma and barriers to mental health care for cannabis users. The participants’ responses challenge public perceptions that cannabis use is harmless and emphasize the value of providing fact-based education for clients and families tailored to address their specific concerns. The findings also challenge the notion that cannabis use is inherently irrational and underscore the importance assessing the individualized psychosocial effects of the drug. Through dispelling myths about cannabis and creating an inclusive therapeutic environment, clients can feel comfortable enough to share their problems, motivations, goals, and appraisals of risk. As these professionals shift clinical and public discourse toward the facts and away from unfounded assumptions, barriers to care for cannabis users who are seeking support (including mental health professionals) can be diminished.

## Conclusion

This study’s findings suggest assessing perceptions of cannabis-related risk among consumers in clinical settings may help reveal latent psychosocial problems. Examining how cannabis affects anxiety, relationships, driving, psychotic symptoms, cognition, and educational and employment functioning can provide a deeper understanding of lived experiences of harm. Helping clients weigh the perceived risks and benefits can set the foundation for therapeutic approaches that address ambivalence and promote recovery. The findings suggest mental health workers may benefit from education regarding the physical health harms associated with this drug. As mental health professionals and services evolve to adapt to the changing health climate, a need remains for improved access to evidence-informed, destigmatized care for people who use cannabis.

## Limitations

This study was limited by time constraints, small sample size, and convenience sampling. Future research using larger samples of professionals and mixed methods approaches may offer more comprehensive understandings of the associations between cannabis use and specific health or clinical outcomes. An analysis of perspectives on stigma, legalization, and barriers to accessing services would have enhanced this study. The findings of this study may not be generalizable to professionals in regions where cannabis is not legal as concerns related to criminal sanctions are likely to be perceived as a risk in jurisdictions where cannabis possession remains a criminal activity. Interviews were conducted prior to widespread awareness of the coronavirus pandemic in Canada and it is unclear how this global crisis may have impacted participant views. This study did not examine choice of cannabis products due to ethical concerns as only legal market products provide validated product information and inquiry into potentially illicit activities was intentionally avoided. There are significant variations in psychoactive effects among various cannabinoids, plant strains, and formulations. Future studies should investigate the perceptions of risk related to specific cannabis products.

## Note

1. Participants were told during the informed consent process that personal disclosure of cannabis-impaired operation of a vehicle (or other behaviors putting themselves or others at risk of imminent harm) may result in a breach of privacy to reduce or eliminate that risk. It is unclear if/how the consent process may have influenced this data.

## References

[bibr1-00220426211032558] Airagnes G. Lemogne C. Meneton P. Plessz M. Goldberg M. Hoertel N., ... Zins M. (2019). Alcohol, tobacco and cannabis use are associated with job loss at follow-up: Findings from the CONSTANCES cohort. PloS One, 14(9), e0222361. 10.1371/journal.pone.0222361 31498849 PMC6733456

[bibr2-00220426211032558] Bradley L. (2018, June 29). The mental health of health care workers. Rehab & Community Care Medicine Magazine. Retrieved from https://www.rehabmagazine.ca/featured-carousel/safeguarding-the-mental-health-of-health-care-workers/

[bibr3-00220426211032558] Caddle (2018). Cannabis stigma—Is there a change on the way? Caddle. Retrieved from https://getcaddle.com/blog/cannabis-stigma-canada/

[bibr4-00220426211032558] CAMH (2020). Addressing stigma: Challenging the stigma associated with mental illness. Addressing Stigma. Retrieved from https://www.camh.ca/en/driving-change/addressing-stigma

[bibr5-00220426211032558] CIHI (2019). Hospital stays for harm caused by substance use among youth age 10 to 24 (p. 22). Canadian Institute for Health Information. Retrieved from https://www.cihi.ca/en/document/hospital-stays-for-harm-caused-by-substance-use-among-youth-age-10-to-24-september-2019

[bibr6-00220426211032558] Coombs R. H. (1996). Addicted health protessionals. Journal of Substance Misuse, 1(4), 187-194. 10.3109/14659899609081954

[bibr7-00220426211032558] Cunningham J. A. Koski-Jännes A. (2019). The last 10 years: Any changes in perceptions of the seriousness of alcohol, cannabis, and substance use in Canada? Substance Abuse Treatment, Prevention, and Policy, 14(1), 54-56. 10.1186/s13011-019-0243-0 PMC6896256 31806035

[bibr8-00220426211032558] Deloitte (2018). A society in transition, an industry ready to bloom [Report] (pp. 1-36). Deloitte. Retrieved from https://www2.deloitte.com/content/dam/Deloitte/ca/Documents/consulting/ca-cannabis-2018-report-en.PDF

[bibr9-00220426211032558] George T. P. Hill K. P. Vaccarino F. J. (2018). Cannabis legalization and psychiatric disorders: Caveat “Hemp-tor”. The Canadian Journal of Psychiatry, 63(7), 447-450. 10.1177/0706743718762387 29482358 PMC6099771

[bibr10-00220426211032558] George-Kosh D. (2020). Ontario online pot purchases jump 600% amid COVID-19 pandemic. Business News Network. Retrieved from https://www.bnnbloomberg.ca/ontario-online-pot-purchases-jump-600-amid-covid-19-pandemic-data-shows-1.1422369

[bibr11-00220426211032558] Ghelani A. (2020). Motives for recreational cannabis use among mental health professionals. Journal of Substance Use, 26, 256-260. 10.1080/14659891.2020.1812124

[bibr12-00220426211032558] Gobbi G. Atkin T. Zytynski T. Wang S. Askari S. Boruff J., ... Mayo N. (2019). Association of cannabis use in adolescence and risk of depression, anxiety, and suicidality in young adulthood. JAMA Psychiatry, 76(4), 426-434. 10.1001/jamapsychiatry.2018.4500 30758486 PMC6450286

[bibr13-00220426211032558] Government of Canada. (2018, November 19). Canadian cannabis survey 2018 summary. Retrieved from https://www.canada.ca/en/services/health/publications/drugs-health-products/canadian-cannabis-survey-2018-summary.html#_Theme_1

[bibr14-00220426211032558] Hathaway A. D. (2003). Cannabis effects and dependency concerns in long-term frequent users: A missing piece of the public health puzzle. Addiction Research & Theory, 11(6), 441-458. 10.1080/1606635021000041807

[bibr15-00220426211032558] Hellemans K. G. C. Wilcox J. Nino J. N. Young M. McQuaid R. J. (2019). Cannabis use, anxiety, and perceptions of risk among Canadian undergraduates: The moderating role of gender. Canadian Journal of Addiction, 10(3), 22-29. 10.1097/CXA.0000000000000059

[bibr16-00220426211032558] Hyman S. M. Sinha R. (2009). Stress-related factors in cannabis use and misuse: Implications for prevention and treatment. Journal of Substance Abuse Treatment, 36(4), 400-413. 10.1016/j.jsat.2008.08.005 19004601 PMC2696937

[bibr17-00220426211032558] Kenna G. A. Baldwin J. N. Trinkoff A. M. Lewis D. C. (2011). Substance use disorders in health care professionals. In Johnson B. A. (Ed.), Addiction medicine: science and practice (pp. 1375-1398). New York: Springer. 10.1007/978-1-4419-0338-9_69

[bibr18-00220426211032558] Kerridge B. T. Mauro P. M. Chou S. P. Saha T. D. Pickering R. P. Fan A. Z., ... Hasin D. S. (2017). Predictors of treatment utilization and barriers to treatment utilization among individuals with lifetime cannabis use disorder in the United States. Drug and Alcohol Dependence, 181, 223-228. 10.1016/j.drugalcdep.2017.09.032 29107786 PMC6310167

[bibr19-00220426211032558] Li S. Seale C. (2007). Learning to do qualitative data analysis: An observational study of doctoral work. Qualitative Health Research, 17(10), 1442-1452. 18000083 10.1177/1049732307306924

[bibr20-00220426211032558] Lynskey M. Hall W. (2000). The effects of adolescent cannabis use on educational attainment: A review. Addiction, 95(11), 1621-1630. 10.1046/j.1360-0443.2000.951116213.x 11219366

[bibr21-00220426211032558] Melchior M. Bolze C. Fombonne E. Surkan P. J. Pryor L. Jauffret-Roustide M. (2017). Early cannabis initiation and educational attainment: Is the association causal? Data from the French TEMPO study. International Journal of Epidemiology, 46(5), 1641-1650. 10.1093/ije/dyx065 28520946

[bibr22-00220426211032558] Moustakas C. E. (1994). Phenomenological research methods (p. xiv). Thousand Oaks, CA: Sage Publications, Inc.

[bibr23-00220426211032558] Osborne G. B. Fogel C. (2008). Understanding the motivations for recreational marijuana use among adult Canadians. Substance Use & Misuse, 43(3–4), 539–572; discussion 573-579, 585–587. 10.1080/10826080701884911 18365950

[bibr24-00220426211032558] Padgett D. K. (2017). Qualitative methods in social work research. New York, NY: SAGE Publications.

[bibr26-00220426211032558] Scott J. (2000). Chapter 9: Rational choice theory. In Browning G. Halcli A. Webster F. (Eds.), Understanding contemporary society: Theories of the present. London: SAGE Publications. 10.4135/9781446218310

[bibr27-00220426211032558] Sinding C. Gray R. Nisker J. (2008). Ethical issues and issues of ethics. In Knowles J. G. Cole A. L. (Eds.), Handbook of the arts in qualitative research: Perspectives, methodologies, examples, and issues. Thousand, CA: SAGE.

[bibr28-00220426211032558] Smith J. A. Jarman M. Osborn M. (1999). Doing interpretative phenomenological analysis. In Murray M. Chamberlain K. (Eds.), Qualitative health psychology (pp. 218-240). London: SAGE Publications.

[bibr29-00220426211032558] Smith J. A. Osborn M. (2008). Four: Interpretative phenomenological analysis. In Smith J. A. (Ed.), Qualitative psychology: A practical guide to research methods (2nd ed., pp. 53-80). London: SAGE Publications.

[bibr30-00220426211032558] Spackman E. Haines-Saah R. Danthurebandara V. Dowsett L. Noseworthy T. Clement F. (2017). Marijuana use and perceptions of risk and harm: A survey among Canadians in 2016. Healthcare Policy, 13(1), 17-27. 10.12927/hcpol.2017.25194 28906233 PMC5595211

[bibr31-00220426211032558] Tasker J. P. (2020). Canadian Medical Association president says “sick” health care system unprepared for a second pandemic wave. CBC. Retrieved from https://www.cbc.ca/news/politics/second-wave-covid-19-sick-health-care-system-1.5577552

[bibr32-00220426211032558] Wallingford S. Konefal S. Young M. (2019). Cannabis use, harms and perceived risks among Canadian students: Technical report (p. 14). Canadian Centre on Substance Use and Addiction. Retrieved from https://www.ccsa.ca/sites/default/files/2019-04/CCSA-Canadian-Students-Cannabis-Harms-Risks-Report-2019-en.pdf

[bibr33-00220426211032558] WHO (2016). World health organization: Health and social effects of nonmedical cannabis use. WHO Library Cataloguing-in-Publication Data. Retrieved from https://www.who.int/substance_abuse/publications/msbcannabis.pdf

[bibr34-00220426211032558] World Economic Forum (2020). U.N. warns of global mental health crisis due to COVID-19 pandemic. World Economic Forum. Retrieved from https://www.weforum.org/agenda/2020/05/united-nations-global-mental-health-crisis-covid19-pandemic/

[bibr35-00220426211032558] Zhang C. Brook J. S. Leukefeld C. G. Brook D. W. (2016). Trajectories of marijuana use from adolescence to adulthood as predictors of unemployment status in the early forties. The American Journal on Addictions, 25(3), 203-209. 10.1111/ajad.12361. 26991779 PMC4809805

